# Changes in intestinal microflora of *Caenorhabditis elegans* following *Bacillus nematocida* B16 infection

**DOI:** 10.1038/srep20178

**Published:** 2016-02-02

**Authors:** Qiuhong Niu, Lin Zhang, Keqin Zhang, Xiaowei Huang, Fengli Hui, Yunchao Kan, Lunguang Yao

**Affiliations:** 1 China-UK-NYNU-RRes Joint Laboratory of Insect Biology, Nanyang Normal University, Nanyang, 473000, P.R. China; 2 Laboratory for Conservation and Utilization of Bio-Resources, Key Laboratory for Microbial Resources of the Ministry of Education, Yunnan University, Kunming 650091, P. R. China

## Abstract

The effect of pathogenic bacteria on a host and its symbiotic microbiota is vital and widespread in the biotic world. The soil-dwelling opportunistic bacterium *Bacillus nematocida* B16 uses a “Trojan horse” mechanism to kill *Caenorhabditis elegans*. The alterations in the intestinal microflora that occur after B16 infection remain unknown. Here, we analyzed the intestinal bacteria presented in normal and infected worms. The gut microbial community experienced a complex change after B16 inoculation, as determined through marked differences in species diversity, structure, distribution and composition between uninfected and infected worms. Regardless of the worm’s origin (i.e., from soil or rotten fruits), the diversity of the intestinal microbiome decreased after infection. *Firmicutes* increased sharply, whereas *Proteobacteria*, *Actinobacteria*, *Cyanobacteria* and *Acidobacteria* decreased to different degrees. *Fusobacteria* was only present 12 h post-infection. After 24 h of infection, 1228 and 1109 bacterial species were identified in the uninfected and infected groups, respectively. The shared species reached 21.97%. The infected group had a greater number of *Bacillus* species but a smaller number of *Pediococcus*, *Halomonas*, *Escherichia* and *Shewanella* species (P < 0.01). Therefore, this study provides the first evaluation of the alterations caused by pathogenic bacteria on symbiotic microbiota using *C. elegans* as the model species.

*Bacillus nematocida* was isolated from a forest soil sample, collected in Yunnan, China, and has been shown to exhibit marked nematotoxic activity against *Caenorhabditis elegans*^1^,^2^,^3^,^4^. This bacterium has also been shown to lure nematodes to their death by a “Trojan horse” mechanism. More specifically, *B. nematocida* lures nematodes by emitting potent volatile organic compounds, and once the bacteria enter the nematode intestine, it secretes two proteases with broad ranges of substrates that preferentially target essential intestinal proteins, leading to the nematode’s death^5^. During this process, the progression of bacterial survival, proliferation and colonization in the host intestine is critical in determining the success or failure of the infection^6^. However, the soil nematode *C. elegans* is a ‘microbivore’ due to its consumption of many species of bacteria. From a microbial perspective, predation avoidance is a highly selected trait that is postulated to be the evolutionary origin for a variety of virulence-related factors^7^. Soil bacteria function in supplying nutrients to and regulating the metabolism of *C. elegans*. Previous studies have identified the normal intestinal flora within the intestines of worms that maintain these stable symbiotic relationships^8^,^9^. Specifically, Félix and Duveau described the presence of bacteria in wild *C. elegans*, but did not identify the species present^8^. Michael Shapira’s research group identified some of the species present in the intestine of *C. elegans* in natural soil, and examined whether these confer resistance to the nematode pathogens *Pseudomonas aeruginosa* and *Enterococcus faecalis*^9^. However, the outcome of the *C. elegans* intestinal flora following colonization by a pathogenic bacterium remains unknown.

Intestinal symbiosis between microorganisms and their host is ubiquitous in the environment. For the hosts, their relationships with intestinal symbiotic microbes range from mutualistic to pathogenic^10^. The gut is home to large communities of microbial flora, including indigenous, opportunistic and pathogenic bacteria. However, symbiotic microbes provide an array of benefits to the host, and the joint efforts between symbiotic microbes and their hosts can prevent the colonization and invasion of pathogenic organisms. The important functions of intestinal microbes in humans have been well characterized^11^,^12^,^13^,^14^,^15^,^16^,^17^,^18^,^19^, but few studies have investigated microbial symbiosis in the intestines of free-living terrestrial nematodes.

The nematode *C. elegans* is involved in complex symbiotic, pathogenic, and predator-prey interactions with its microbial community. Furthermore, *C. elegans* has been an attractive model organism for studying host-pathogen interactions since the 1970s^7^. Their intestine has high metabolic activity and functions similar to those of the fat body in flies and the liver in mammals^20^. Although *C. elegans* is a common model organism in laboratory settings, few researchers have paid attention to its growth status under natural conditions. Additionally, the mechanism underlying intestinal changes in microflora during colonization by pathogenic bacteria remains elusive.

In this study, we examined and identified the “normal” intestinal microflora of adult *C. elegans* as well as the changes that occur after infection with *Bacillus nematocida* B16. This manuscript provides novel insights that may help improve the understanding of host–microbe interactions.

## Materials and Methods

### Strains, worms and culture conditions

A specimen of *Bacillus nematocida* with significant nematotoxic activities^2^, designated strain B16, was deposited at the China General Microbiological Culture Collection Center (CGMCC, catalogue 1128). This strain is typically incubated on Luria-Bertani (LB) medium at 37 °C and used as an opportunistic pathogen.

The growth and synchronization of worm cultures were performed as follows: First, the worm, *C. elegans* N2 strain, was grown on solid standard nematode growth medium (NGM) plates at 25 °C and fed *E. coli* OP50 using water-soluble cholesterol. The worms were then separated from the bacteria by sedimentation and sucrose flotation as previously described^21^. Eggs were obtained by incubating mixed-stage populations with alkaline hypochlorite^22^. Synchronous cultures were achieved by allowing the purified eggs to hatch overnight in S medium without bacteria^21^, and the larval stage 1 (L1) animals were then washed free of the dauer pheromone and diluted to 10^4^ worms/ml for soil exposure.

Triplicate soil samples were collected in sterile conical tubes at the vertex of a triangle with an edge distance of 1 m located at the rhizosphere of wild orange trees at the Baotianman Nature Reserve. At each sampling site, we collected all the lower (18–20 cm) layers of the soil to ensure that the collected soils were homogeneous. Typical soil is important ecologically because this natural soil is similar to the temperate soil environments from where these worms were originally collected. The soil samples were stored at 4 °C until use. Just prior to use, the soil samples were weighed and placed into Petri dishes. Each gram of soil was added to 1 ml of the worm slurry and incubated at 25 °C for 72 h until formation of the adult stage was achieved. Other nematodes in the soil samples were excluded due to their different morphological characteristics. The different stages of *C. elegans* were verified by visual inspection under a microscope. Adult worms of the same size were harvested and selected for the subsequent experiments.

Other batches of worms were isolated from rotten fruits using the same method.

### Infection assay

The nematodes were separated using the Baerman funnel technique^23^ and then washed three times with M9 buffer. An aqueous suspension of nematodes was prepared to obtain a working stock. The infection of the worms by *B. nematocida* B16 was performed according to the ‘feeding transfer’ experiments described by Rosen *et al*.^6^,^24^ with minor modifications. Briefly, pieces of autoclaved cellophane paper were used to cover NG agar medium. Bacteria were inoculated onto the cellophane paper and incubated at 37 °C for 3 d. Two hundred microliters of the nematode suspensions (each containing approximately 150 worms) were then placed on *B. nematocida* B16 lawns for 4 h. The worms were then removed from the plates, washed twice in M9 buffer, and transferred to plates (prepared as above) containing *E. coli* for 3 days. During this period, the worms were transferred to new NGM plates every 12 h. The nematodes were considered dead when no movement was observed under a light-dissecting microscope, and when gentle tapping of the nematodes using a stick did not result in movement. The number of deceased worms in each group was counted every 12 h. The worms that died as a result of adhering to the plate wall were excluded from the analysis. Worms that were seeded only on *E. coli* lawns throughout the duration of the experiment but were still transferred every 12 h served as negative controls. The experiments were performed with three parallel replicates and repeated three times. Finally, the worms were washed again with sterile water and used for the subsequent experiments.

### Intestinal histopathology and electron microscopy

A total of 20–50 worms were randomly selected, surface-sterilized by soaking in a 1% mercuric chloride and 2% antibiotic mixture containing streptomycin sulfate and gentamicin for 1 h, and then cultured on nutrient and oligotrophic agar plates to confirm successful surface sterilization (this was confirmed if 0 cfu was found). The nematode samples were prepared as described in our previous paper^5^, and changes in the nematode intestines were observed through light microscopy and transmission electron microscopy (TEM).

### Worm collection and bacterial DNA extraction

Approximately 5,000 worms from the uninfected controls and the 6- to 24-h-infected worms were collected from the plates and treated based on the method described in the literature^25^. The worms were washed with 100 mM levamisole and incubated in M9 containing 100 mM levamisole and 100 mg/ml gentamicin. After incubation, the adult worms were washed with a levamisole solution to remove the gentamicin and homogenized with M9 containing 1% Triton X-100 to extract the total DNA of the intestinal microbes by grounding with a 1-ml micro-dismembrator (Wheaton).

DNA extraction was performed as previously described^26^. The QIAamp DNA Stool Mini Kit (Qiagen, West Sussex, United Kingdom) was used according to the manufacturer’s instructions. The genomic DNA was evaluated with a NanoDrop spectrophotometer, using an A260/A280 ratio between 1.8 and 2.0 as a criterion for quality control.

### Amplification of 16S rDNA V4 hypervariable regions

Two universal primers (forward: 5′-AYT GGGYDTAAAGNG-3′, reverse: 5′-TACNVGGGTATCTAATCC-3′) were used to amplify the V4 16S rDNA hypervariable regions. PCR amplification was performed: using 25 cycles of 94 °C for 30 s, 50 °C for 30 s, and 72 °C for 30 s. The PCR products were separated on a 1% agarose gel, purified using the QIAquick Gel Extraction Kit and sequenced by Illumina MiSeq.

### Bioinformatic and statistical analyses

QIME software was used for the filtering analysis^27^. Low-quality sequences were truncated, and the ends of the corresponding sequences were connected using Flash software^28^. High-quality sequences were classified into multiple operational taxonomic units (OTUs) with UCLUST software based on a similarity greater than 97%^29^. The mean lengths of the sequence reads were classified into different taxonomic categories using MGRAST^30^. The taxon abundance of each sample was categorized with the RDP classifier^31^. The diversity of the two worm groups was analyzed using the Mother software according to the species richness in the list of OTUs. The phylotype richness was evaluated by the Chao/Ace calculation, and the Shannon index of diversity was used as an estimator of both the richness and community evenness. The extent of similarities between different ecosystems was investigated using the UniFrac β-diversity.

Statistical analyses were based on the number of sequence reads that belonged to each taxa and were performed as described by Li^32^. SPSS 16.0 software was used for the statistical analyses. Significant differences in basal characteristics between the groups were calculated by one-way analysis of variance and Student’s *t* test for continuous variables. *P* < 0.05 was considered statistically significant.

## Results

### Infection results via the ‘feeding transfer’ test

The results from the ‘feeding transfer’ experiments, which were designed to demonstrate the pathogenic consequences of *B. nematocida*, are shown in Table 1. Consistent with the results described in our previous articles^4^,^6^, almost 80% of the infected *C. elegans* tested died over the course of 3 d when the worms were placed on a *B. nematocida* B16 lawn for 4 h before being transferred to a lawn of *E. coli*. Conversely, only 15% of the negative-control worms, which were only fed *E. coli*, died. These results confirm that only a small inoculum of B16 can proliferate in the *C. elegans* intestine, become the dominant bacterium, and ultimately colonize and establish a persistent infection. Moreover, we found that the cuticles of the majority of the tested soil worms were destroyed after 24 h of infection, which resulted in the inability to wash off cuticle-associated microbes. Therefore, we chose 24 h after the shift from B16 to OP50 as the time point for our subsequent study.

### Observation of intestinal bacteria

**Figure 1:**
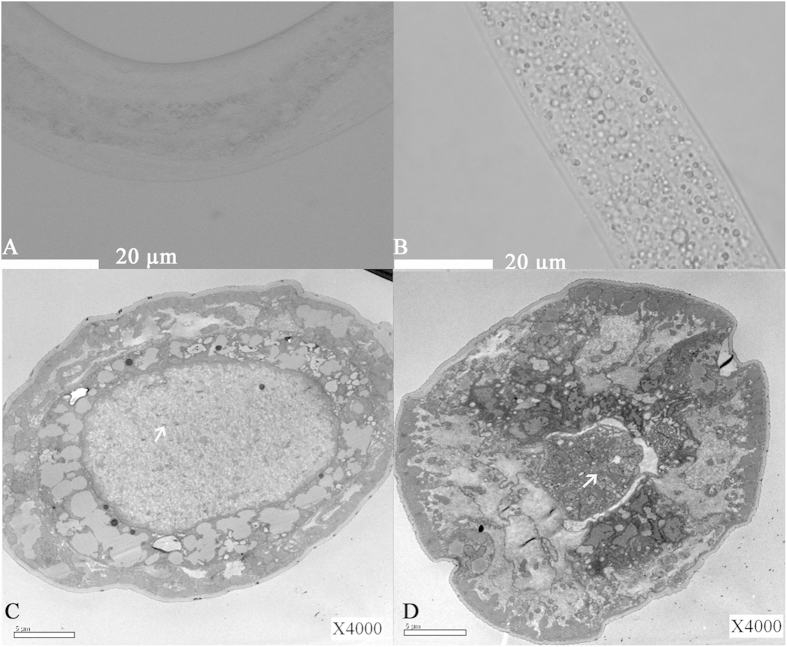
Structural analysis of *C. elegans* intestines and detailed pictures of endophytic bacteria obtained via TEM. Photographs of *C. elegans* originating from soil. (**A**) Light microscopy showed that the worms were alive and that their intestines were normal after feeding on *E. coli* for 48 h; (**B**) Light microscopy showed that the worms were dead and their intestines were disorganized after feeding on *B. nematocida* strain B16g-1 for 4 h prior to transfer to *E. coli* plates for 24 h; (**C**) Cross-section of uninfected worms (4000-fold amplification); (**D**) Cross-section of 24-h-infected worms (4000-fold amplification). Symbiotic microbiota are indicated with white arrows in (**C**,**D**).

As described previously^5^, uninfected soil worms fed *E. coli* presented intact and complete gut organization (Figs 1A,C), whereas worms infected for 24 h revealed disordered and loose intestinal structures under both an optical microscope and TEM (Figs 1B,D). Furthermore, no bacterial colonies were observed when the surface-sterilized solution was spread onto LB and oligotrophic agar mediums, a result that confirmed successful surface sterilization. In contrast, intestinal bacteria were clearly observed by TEM in the intestines of both the uninfected and 24-h-infected groups. In the uninfected group, most of the cocci and some of the bacilli were observed in the intestines (Fig. 1C), whereas the majority of bacilli were found in the intestines of the 24-h-infected group (Fig. 1D). The sizes of the intestinal bacteria were approximately (1.1 × 0.3) μm–(2.5 × 0.3) μm.

### Original data and operational taxonomic unit analysis

A total of 195, 125 V4 16S rDNA sequence reads from the worms originating from the soil, including the uninfected controls and the groups infected for 12 h and 24 h, with an average of 70,322 sequence reads for each sample, were used in this analysis. The mean length of the sequence reads reached 255.79 bp.

**Figure 2:**
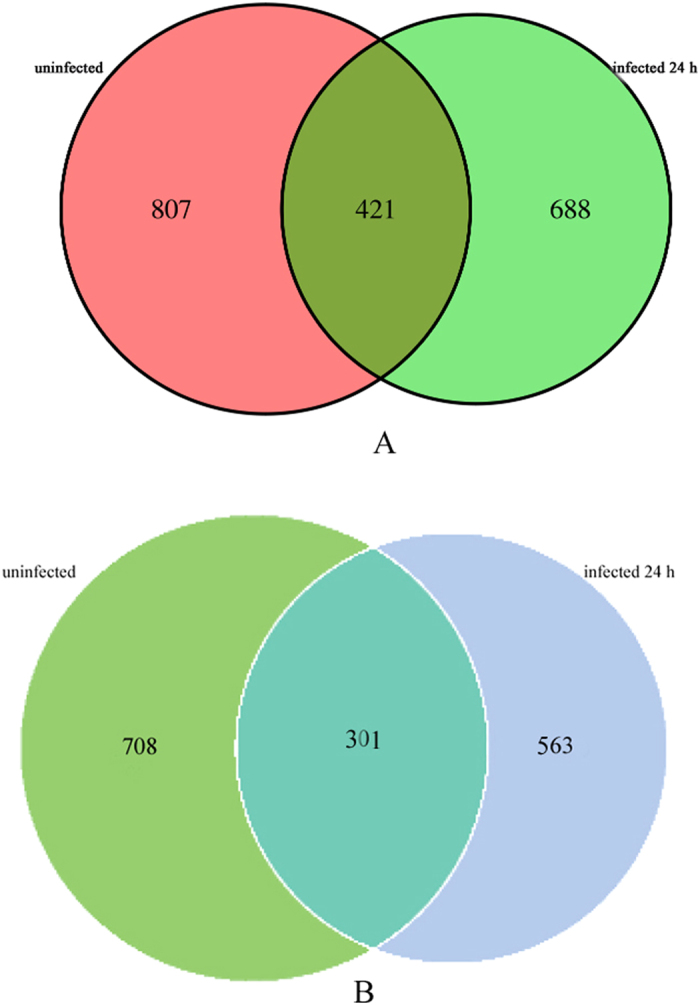
Venn diagram comparing the uninfected and 24-h-infected groups. (**A**) Data for worms from soil; (**B**) Data for worms from rotten fruits.

The taxon abundance of each sample was categorized into 16 phyla, 26 classes, 46 orders, 71 families and 106 genera. Up to 1228, 1202 and 1109 species were found in the uninfected controls, 12-h-infected and 24-h-infected groups, respectively. In addition, 1989 species were identified across the three groups, and the richness of the shared species between the uninfected controls and the 24-h-infected group was 421, which accounted for 21.97% (Fig. 2A).

The analysis performed with worms collected from rotten fruits revealed 1009 and 864 species in the uninfected and 24-h-infected groups, respectively, with 301 species, corresponding to 19.15%, shared between the two groups (Fig. 2B).

### Variance analysis of species abundance

**Figure 3:**
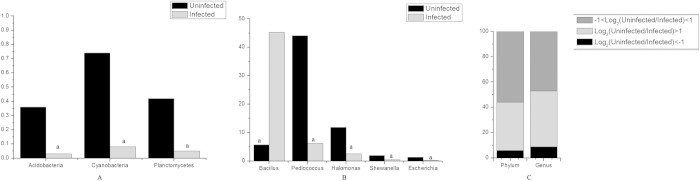
Statistical results of the comparison of the significant microflora of soil worms between the uninfected and 24-h-infected groups. (**A**) At the phylum level, *Actinobacteria*, *Cyanobacteria* and *Planctomycetes* differed significantly between the two samples. (**B**) At the genus level, *Bacillus, Pediococcus, Halomonas, Shewanella* and *Escherichia* differed significantly between the two groups. The vertical axis represents the sequence reads. (**C**) Percentage of phylum and genus levels. ^a^*P* < 0.05.

The subsequent analysis of the soil-originating worms revealed a total of 17 phyla and 110 genera in the three samples. The 24-h-infected group had a higher number of *Firmicutes* species [log_2_(uninfected/infected 24 h) < −1] and presented a two-fold decrease in *Actinobacteria*, *Cyanobacteria*, *Planctomycetes*, *Acidobacteria*, *Chloroflexi*, and *Bacteroidetes* species [log_2_(uninfected/infected 24 h) > 1] relative to the uninfected control group. A significant difference in the number of *Acidobacteria, Cyanobacteria* and *Planctomycetes* species was found between the two groups (*P* < 0.05) (Table 2, Fig. 3A). Compared with the uninfected control group, the 24-h-infected group presented more than a two-fold increase in species from nine genera, including *Bacillus*, *Stenotrophomonas, Rhizobium*, *Agrobacterium*, *Pseudonocardia, Phenylobacterium, Prosthecobacter, Streptococcus* and *Pseudoxanthomonas* and more than two-fold decreases in species from 47 genera, including *Pediococcus*, *Halomonas*, *Shewanella*, *Sphingomonas*, *Escherichia* and *Acinetobacter*. Significant differences in the number of species of *Bacillus, Pediococcus, Halomonas, Shewanella* and *Escherichia* were found between the two groups (*P* < 0.05) (Table 3, Fig. 3B). The percentage of species that were found in numbers at least two-fold higher in the 24-h-infected group compared with the uninfected control group was 6% at the phylum level and 9% at the genus level, whereas the corresponding percentage of species that were found in number at least two-fold lower was 38% at the phylum level and 44% at the genus level. Conversely, the percentage of species that presented changes of less than twofold was 56% at the phylum level and 47% at the genus level (Fig. 3C).

### Single-sample species distribution

**Figure 4:**
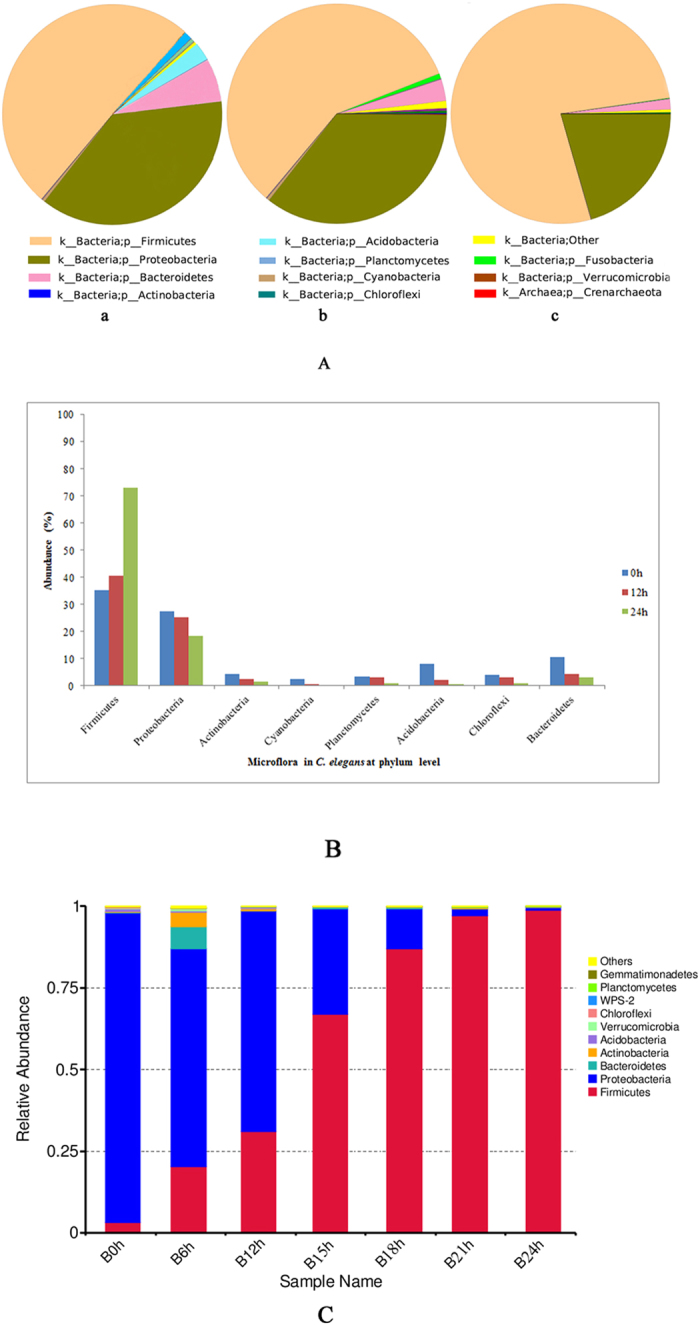
(**A**) Pie charts of the bacterial distribution at the phylum level in normal and infected worms from soil. a. Uninfected controls; b. 12 h after infection and shifting from B16 to OP50; c. 24 h after infection and shifting from B16 to OP50. K and P represent the kingdom and phylum, respectively. (**B**) Changes in the major bacterial populations of normal and infected worms from soil at the phylum level. (**C**) Column diagram of the bacterial distribution at the phylum level in normal and infected worms from rotten fruit.

**Figure 5:**
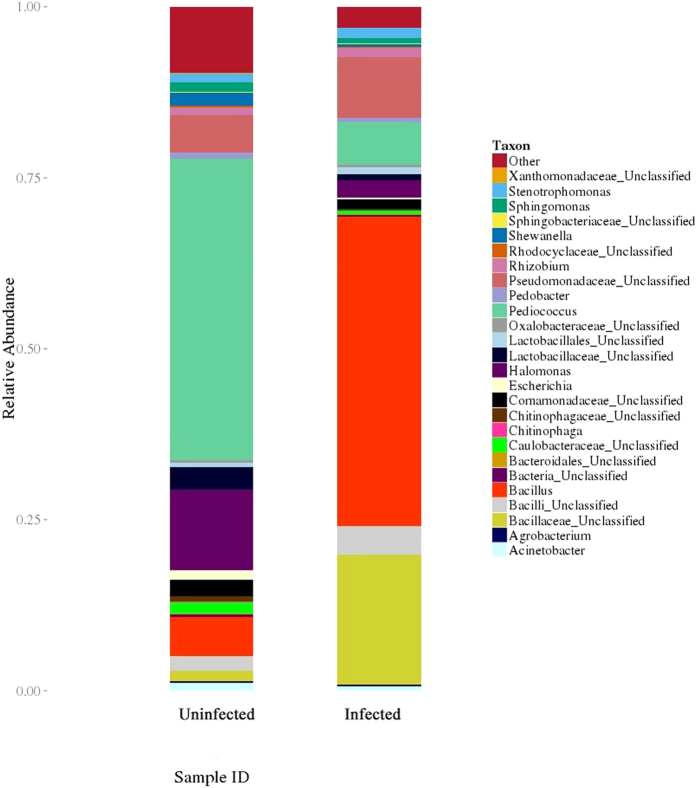
Comparison of species abundance between the normal and 24-h-infected worms originating from soil at the genus level.

A pie chart of each species distribution was recorded, providing information on classification and abundance within the OTU list. At the phylum level, a total of 12 phyla were found in the three groups from the soil. In addition to these, *Crenarchaeota*, *Chlamydiae*, *Elusimicrobia* and *Chlorobi* were also found in the uninfected control group, but no other unique phyla were found in the infected groups. The percent distributions of the microbial community in normal and infected nematodes are summarized in Fig. 4. Compared with the uninfected controls, only *Firmicutes* increased sharply in infected worms, whereas *Proteobacteria*, *Actinobacteria*, *Cyanobacteria*, *Planctomycetes*, *Acidobacteria*, *Chloroflexi* and *Bacteroidetes* decreased to different degrees. The same results were obtained for nematodes originating from rotten fruits (Fig. 4C). Furthermore, *Fusobacteria* was present at 12 h post-infection but absent after 24 h of infection. Additionally, some unclassified bacteria were slightly increased at 12 h but decreased at 24 h. We inferred that inoculation with *B. nematocida* caused some indigenous microflora to increase in number. As a result, these microbes may prevent infection by the pathogen B16, allowing *B. nematocida* to out-compete these intestinal microflora and colonize successfully. The results showed that the microbiome of *C. elegans* experiences a complex change when the nematodes are infected with the opportunistic pathogen *B. nematocida* B16. The analysis compared the species abundance between the uninfected and 24-h-infected groups at the genus level, and the results are shown in Fig. 5. At the genus level, the percent distributions of the microbial community in the uninfected and 24-h-infected nematodes were 6 and 45 for *Bacillus*, 44 and 6 for *Pediococcus*, 12 and 3 for *Halomonas*, 2 and 0.4 for *Shewanella*, and 1 and 0.2 for *Escherichia*, respectively.

### Alpha and β-diversity analysis

The uninfected group had both a higher richness index (Chao, 4663.2 and ACE, 7891.3) and a higher diversity index (4.49) than the 24-h-infected group (Chao, 3352.8, ACE, 6355.9 and Shannon, 4.01). These results reveal that the uninfected group exhibits higher levels of biodiversity and unevenness estimations than the 24-h-infected group. The range found was reflected by Good’s coverage in this study and was between 90.7% and 91.9% for the data sets with a 97% similarity level (Table 4).

**Figure 6:**
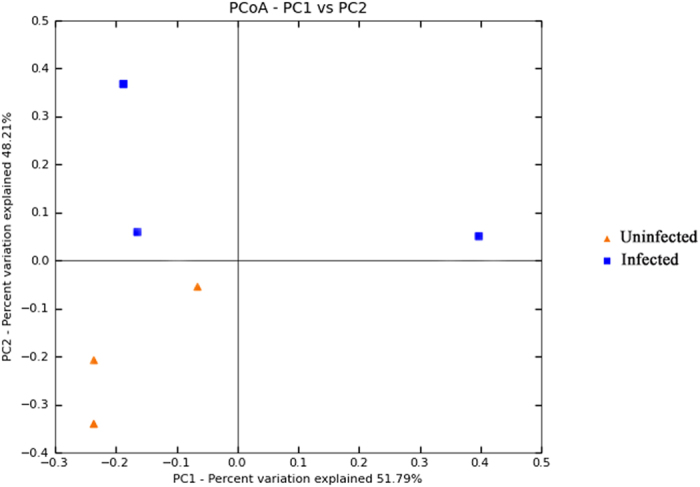
Changes in microbial diversity in the guts of normal and 24-h-infected worms from soil. Six samples were analyzed, and the clustering of the microbial communities was performed using the PCoA of the weighted UniFrac matrix. The percentage of variation indicated by the principal coordinates is shown on the axes. Color coding: blue, samples from the 24-h-infected group; red, samples from the uninfected control. PCoA: Principal co-ordinate analysis.

According to the UniFrac PCoA analysis of 1916 OTUs, a clear separation was found between the uninfected and 24-h-infected worms originating from soil (Fig. 6). The percentages of variation detected by PC1 and PC2 were 51.79% and 48.21%, respectively. The 24-h-infected group was well separated from the uninfected group according to the weighted analysis.

## Discussion

The analysis of the gut microbiome in animals is currently a hot topic because the gut microbiota status is a determinant of the host’s health^33^. The gut microbial populations influence the host’s conditions, and inversely, the host’s conditions also influence the microbial populations. Numerous studies have shown that the density, composition, and complexity of the gut microbiota have strong effects on pathogen colonization, immune responses, and pathogen clearance^34^,^35^. However, the alteration of the density, composition and complexity of the gut microbiota after the introduction of a pathogen into animals is largely unknown. Therefore, searching for a rational model organism that can be utilized to investigate such changes is scientifically valuable.

The nematode *C. elegans* is genetically tractable and a major model organism for studies of microbial pathogenesis and human health. It has been found that bacteria accumulate in the *C. elegans* intestine with aging and that the intestinal bacterial load is regulated by gut immunity and influences longevity^36^. In fact, *C. elegans* are involved in complex symbiotic, pathogenic, and predator-prey interactions with their microbial community. Thus, the bacteria-eating *C. elegans* provide an opportunity to dissect bacteria-host interactions. It is reasonable to consider that *C. elegans* can be attacked by natural pathogenic bacteria from their ecological environment. Therefore, *C. elegans* combined with one microbial species is an excellent, defined model system for investigating the mechanisms underlying host–microbiota interactions^33^ and is particularly suited to the study of intestinal microflora-pathogen interactions. Our research group identified *B. nematocida* B16, an opportunistic bacterial pathogen, in a soil sample from Yunnan Province in China^1^. As previously mentioned, this bacterial-mediated killing of *C. elegans* typically correlates to the accumulation of bacteria in the intestinal lumen^5^,^6^. When *C. elegans* feed on non-pathogenic *E. coli*, few intact bacteria are found in the intestine; however, when feeding on B16, large quantities of intact pathogen cells accumulate in the intestinal lumen, which can become grossly distended^6^. Prior to this study, little was known about the natural intestinal flora of healthy C*. elegans*, and nothing was known regarding changes in the intestinal flora induced by a bacterial pathogen.

Scientists have recently evaluated the abundance and biodiversity of intestinal bacteria under healthy and diseased conditions through high-throughput sequencing, which is the most common method for analyzing the diversity of environmental microbes, including uncultured microorganisms and trace amounts of bacteria. An imbalance in the gut microflora is evident in diabetes^37^, cancer^38^,^39^ and obese patients^40^. It was recently reported that three genera, *Ochrobactrum*, *Pedobacter*, and *Chitinophaga*, are found at high levels in the nematode *Acrobeloides maximus* living in soil. The putative symbionts *Ochrobactrum* and *Pedobacter* are maintained in nematode guts^10^. In this study, we clearly observed the presence of symbiotic bacteria in the intestines of *C. elegans* cultured from soil. We also compared the microbiome of the worm intestines in the presence and absence of the pathogenic bacteria *B. nematocida* B16 through 16S rDNA-based molecular sequencing and found significant differences in the species and distribution between the control worms and those infected with the pathogen *B. nematocida* B16 for 24 h. The sequencing results indicate the presence of 1228 and 1109 species in the control and infected groups, respectively, revealing lower values of biodiversity and species richness in the infected group relative to the control group. However, there are factors that may influence the results. Figure 2 shows 688 bacterial species that are specific to the B16-infected worms, which may be explained as follows: We attempted to ensure consistency of the worms between the normal and infected groups across the whole experimental procedure, such as utilizing the same soil methods and nematodes with the same size and age, which suggests that the only difference between the two worm populations was the introduction of B16 into one set of worms. However, we would not rule out the possibility that individual differences between the worms could have affected the species of bacteria in their intestines. In addition, the sensitivity of the high-throughput 16S rDNA sequencing technique influences the experimental result. Some bacteria were recovered in miniscule amounts that could not be detected from the uninfected controls but changed to become the predominant microflora after infection with *B. nematocida* B16. To verify the reproducibility of our results, we also performed the same experiments using *C. elegans* worms from rotten fruits. The worms from rotten fruits contained many *Pseudomonas* species, *Stenotrophomonas* species, *Bacillus* species and some *Enterobacteria*, presenting slight differences compared with the worms from the soil, which also contained *Pediococcus* and *Halomonas* species. These results indicate that nematodes from different origins are likely to contain very different sets of bacteria. However, additional data showed that similar reductions in bacterial diversity occurred in the worm samples from soil and rotten fruit due to infection. Moreover, the numbers of *Firmicutes* increased and the numbers of *Proteobacteria* decreased during the infection process, which is consistent with the results obtained using worms from soil. Therefore, our conclusions are generally applicable.

Compared with the control group, the infected group had a significantly greater number of *Bacillus* species but lower numbers of *Pediococcus*, *Halomonas*, *Shewanella* and *Escherichia* at the genus level, as determined by the abundance difference analysis. These results are consistent with our previous reports on the proliferation of *B. nematocida* in the intestines of *C. elegans*^6^ but slightly different from the findings reported by Baquiran *et al*. in 2013^10^. *Pediococcus*, *Halomonas*, *Escherichia* and *Sphingomonas*, which are the primary unique bacteria in healthy individuals, function in preventing the invasion of pathogenic bacteria. Wang *et al*. reported that *Pediococcus acidilactici* in the vaginas of dairy cows produced the bacteriocin pediocin AcH/PA-1 to combat uterine infections^41^. Wang and Zhou identified a *Halomonas* GY1 from the Huanghai soil in Lianyungang, China, which could inhibit Gram-positive and Gram-negative bacteria depending on the polysaccharide involved^42^. Da Re *et al*. studied commensal *Escherichia coli* genes involved in biofilm resistance to pathogen colonization^43^. In addition, *Sphingomonas* strains have been reported to protect *Arabidopsis thaliana* against *Pseudomonas* colonization^44^,^45^. According to the studies referenced above, we deduced that the intestinal microflora in *C. elegans* play a role in resisting pathogen colonization and that *B. nematocida* B16 was ultimately able to overcome this microflora. This study provides the first description of the characteristics of the intestinal microflora in healthy *C. elegans* and in worms infected with *B. nematocida*. Therefore, this study identified the commensal bacteria in *C. elegans* and assessed the changes that occurred after an infection with a pathogenic bacterium. Our results may lead to a better understanding of the mechanism and evolution of the mutualistic relationship between a host and its gut microbial community based on the nematode’s molecular genetics. An analysis of the variety of intestinal bacteria will lay foundations for studying the functions of colonization-resisting pathogens as well as the physical and ecological roles of nematodes, which will aid the understanding of pathogen–commensal bacteria–host interactions. Moreover, further analysis of the microflora of infected worms will assist the understanding of the role of microflora in establishing colonization resistance in its natural habitat.

## Additional Information

**How to cite this article**: Niu, Q. *et al*. Changes in intestinal microflora of *Caenorhabditis elegans* following *Bacillus nematocida* B16 infection. *Sci. Rep*. **6**, 20178; doi: 10.1038/srep20178 (2016).

## Figures and Tables

**Table 1:** Killing of soil *C. elegans* with *B. nematocida* during ‘feeding transfer’ experiments.

Samples	Nematode mortality (%) (SD)					
	12 h	24 h	36 h	48 h	60 h	72 h
B16 (4 h) + *E. coli*	20(1.7)	40(2.2)	45(2.0)	50(2.4)	70(3.0)	80(3.2)
*E. coli*	5(0.2)	5(0.2)	8(0.3)	10(0.3)	12(0.4)	15(0.3)
Blank medium	5(0.2)	5(0.3)	8(0.4)	10(0.4)	12(0.3)	15(0.4)

**Table 2:** Variance analysis of bacterial abundance at the phylum level.

Log_2_(uninfected/infected 24 h)	Taxon
Log_2_(uninfected/infected 24 h) < −1	Bacteria; Firmicutes
Log_2_(uninfected/infected 24 h) > 1	Bacteria; Actinobacteria
	Bacteria; Cyanobacteria*
	Bacteria; Planctomycetes*
	Bacteria; Acidobacteria*
	Bacteria; Chloroflexi
	Bacteria; Bacteroidetes

For bacteria; *Acidobacteria*; ^*^P < 0.05 *vs*. control (*P* = 0.0096).

For bacteria; *Cyanobacteria*; ^*^P < 0.05 *vs*. control (*P* = 0.0201).

For bacteria; *Planctomycetes*; ^*^P < 0.05 *vs*. control (*P* = 0.0183).

**Table 3:** Variance analysis of bacterial abundance between uninfected and infected groups at 24 h for the genus level.

Log_2_(uninfected/infected)	Taxon
Log_2_(uninfected/infected) < −1	Bacteria;Firmicutes;Bacilli;Bacillales;Bacillaceae;Bacillus
9	Bacteria;Proteobacteria;Gammaproteobacteria;Xanthomonadales;Xanthomonadaceae;Stenotrophomonas
	Bacteria;Proteobacteria;Alphaproteobacteria;Rhizobiales;Rhizobiaceae;Rhizobium
	Bacteria;Proteobacteria;Alphaproteobacteria;Rhizobiales;Rhizobiaceae;Agrobacterium
	Bacteria;Actinobacteria;Actinobacteria;Actinomycetales;Pseudonocardiaceae;Pseudonocardia
	Bacteria;Proteobacteria;Alphaproteobacteria;Caulobacterales;Caulobacteraceae;Phenylobacterium
	Bacteria;Verrucomicrobia;Verrucomicrobiae;Verrucomicrobiales;Verrucomicrobiaceae;Prosthecobacter
	Bacteria;Firmicutes;Bacilli;Lactobacillales;Streptococcaceae;Streptococcus
	Bacteria;Proteobacteria;Gammaproteobacteria;Xanthomonadales;Xanthomonadaceae;Pseudoxanthomonas
Log_2_(uninfected/infected) > 1	Bacteria;Firmicutes;Bacilli;Lactobacillales;Lactobacillaceae;Pediococcus
47	Bacteria;Proteobacteria;Gammaproteobacteria;Oceanospirillales;Halomonadaceae;Halomonas
	Bacteria;Proteobacteria;Gammaproteobacteria;Alteromonadales;Shewanellaceae;Shewanella
	Bacteria;Proteobacteria;Gammaproteobacteria;Enterobacteriales;Enterobacteriaceae;Escherichia
	Bacteria;Proteobacteria;Alphaproteobacteria;Sphingomonadales;Sphingomonadaceae;Sphingomonas
	Bacteria;Proteobacteria;Gammaproteobacteria;Pseudomonadales;Moraxellaceae;Acinetobacter
	Bacteria;Bacteroidetes;Sphingobacteriia;Sphingobacteriales;Sphingobacteriaceae;Pedobacter
	Bacteria;Proteobacteria;Alphaproteobacteria;Rhizobiales;Brucellaceae;Ochrobactrum
	Bacteria;Proteobacteria;Gammaproteobacteria;Xanthomonadales;Sinobacteraceae;Nevskia
	Bacteria;Planctomycetes;Planctomycetia;Gemmatales;Gemmataceae;Gemmata
	Bacteria;Proteobacteria;Alphaproteobacteria;Rhizobiales;Bradyrhizobiaceae;Bradyrhizobium
	Bacteria;Proteobacteria;Alphaproteobacteria;Rhizobiales;Hyphomicrobiaceae;Rhodoplanes
	Bacteria;Acidobacteria;Solibacteres;Solibacterales;Solibacteraceae;Candidatus Solibacter
	Bacteria;Bacteroidetes;Bacteroidia;Bacteroidales;Bacteroidaceae;Bacteroides
	Bacteria;Proteobacteria;Alphaproteobacteria;Sphingomonadales;Sphingomonadaceae;Novosphingobium
	Bacteria;Bacteroidetes;Bacteroidia;Bacteroidales;Prevotellaceae;Prevotella
	Bacteria;Proteobacteria;Alphaproteobacteria;Caulobacterales;Caulobacteraceae;Caulobacter
	Bacteria;Proteobacteria;Alphaproteobacteria;Rhizobiales;Methylobacteriaceae;Methylobacterium
	Bacteria;Actinobacteria;Actinobacteria;Actinomycetales;Promicromonosporaceae;Cellulosimicrobium
	Bacteria;Actinobacteria;Actinobacteria;Actinomycetales;Corynebacteriaceae;Corynebacterium
	Bacteria;Proteobacteria;Gammaproteobacteria;Pseudomonadales;Moraxellaceae;Enhydrobacter
	Bacteria;Firmicutes;Bacilli;Bacillales;Staphylococcaceae;Staphylococcus
	Bacteria;Proteobacteria;Alphaproteobacteria;Rhodobacterales;Rhodobacteraceae;Paracoccus
	Bacteria;Actinobacteria;Rubrobacteria;Rubrobacterales;Rubrobacteraceae;Rubrobacter
	Bacteria;Planctomycetes;Planctomycetia;Planctomycetales;Planctomycetaceae;Planctomyces
	Bacteria;Proteobacteria;Gammaproteobacteria;Xanthomonadales;Xanthomonadaceae;Luteimonas
	Bacteria;Bacteroidetes;Bacteroidia;Bacteroidales;Porphyromonadaceae;Parabacteroides
	Bacteria;Proteobacteria;Betaproteobacteria;Burkholderiales;Oxalobacteraceae;Ralstonia
	Bacteria;Proteobacteria;Deltaproteobacteria;Bdellovibrionales;Bdellovibrionaceae;Bdellovibrio
	Bacteria;Proteobacteria;Alphaproteobacteria;Sphingomonadales;Sphingomonadaceae;Sphingobium
	Bacteria;Actinobacteria;Actinobacteria;Actinomycetales;Nocardioidaceae;Aeromicrobium
	Bacteria;Bacteroidetes;Sphingobacteriia;Sphingobacteriales;Flexibacteraceae;Dyadobacter
	Bacteria;Bacteroidetes;Bacteroidia;Bacteroidales;Paraprevotellaceae;Prevotella
	Bacteria;Proteobacteria;Gammaproteobacteria;Legionellales;Legionellaceae;Legionella
	Bacteria;Proteobacteria;Gammaproteobacteria;Enterobacteriales;Enterobacteriaceae;Erwinia
	Bacteria;Proteobacteria;Deltaproteobacteria;Desulfovibrionales;Desulfovibrionaceae;Bilophila
	Bacteria;Proteobacteria;Betaproteobacteria;Burkholderiales;Alcaligenaceae;Sutterella
	Bacteria;Firmicutes;Erysipelotrichi;Erysipelotrichales;Coprobacillaceae;Catenibacterium
	Bacteria;Firmicutes;Clostridia;Clostridiales;Ruminococcaceae;Faecalibacterium
	Bacteria;Proteobacteria;Gammaproteobacteria;Legionellales;Coxiellaceae;Aquicella
	Bacteria;Firmicutes;Clostridia;Clostridiales;Lachnospiraceae;Ruminococcus
	Bacteria;Actinobacteria;Actinobacteria;Actinomycetales;Micrococcaceae;Microbispora
	Bacteria;Proteobacteria;Alphaproteobacteria;Rhizobiales;Bradyrhizobiaceae;Bosea
	Bacteria;Firmicutes;Clostridia;Clostridiales;Veillonellaceae;Megasphaera
	Bacteria;Firmicutes;Bacilli;Bacillales;Planococcaceae;Sporosarcina
	Bacteria;Actinobacteria;Actinobacteria;Actinomycetales;Brevibacteriaceae;Brevibacterium
	Bacteria;Bacteroidetes;Sphingobacteriia;Sphingobacteriales;Sphingobacteriaceae;Sphingobacterium

**Table 4:** Estimation of diversity within the normal and 24-h-infected worms from soil.

Group	Chao	Ace	Shannon	Coverage
Normal	4663.2	7891.3	4.49	0.907
Infection	3352.8	6355.9	4.01	0.919
